# Oral microbial profiles in young adults with cannabis use disorder

**DOI:** 10.1016/j.drugalcdep.2025.112822

**Published:** 2025-08-07

**Authors:** Brittney D. Browning, Rachel L. Tomko, Anna E. Kirkland, Rachel Visontay, Pamela L. Ferguson, Alexander V. Alekseyenko, Melinda A. Engevik, Louise Mewton, Lindsay M. Squeglia

**Affiliations:** a Department of Psychiatry and Behavioral Sciences, Medical University of South Carolina, Charleston, SC, USA; b Hollings Cancer Center, Medical University of South Carolina, Charleston, SC, USA; c The Matilda Centre for Research in Mental Health and Substance Use, University of Syndey, Sydney, Australia; d Department of Public Health Sciences, Medical University of South Carolina, Charleston, SC, USA; e Department of Regenerative Medicine, Medical University of South Carolina, Charleston, SC, USA

**Keywords:** Cannabis, Microbiome, Young adult, Substance use

## Abstract

**Background:**

With increasing legalization and rising potency of cannabis products, cannabis use disorder (CUD) is a growing public health concern, particularly among young adults, who have the highest rates of CUD. While substance use is known to be associated with the oral microbiome, the impact of CUD remains understudied. Given the oral microbiome’s role in overall health, identifying microbial signatures associated with CUD, relative to other substance use disorders (SUDs), may provide insight into its biological mechanisms and potential therapeutic targets.

**Methods:**

Saliva samples were collected from young adults (ages 18–25; N = 192) with CUD (n = 129) and non-CUD SUD (n = 63). The non-CUD SUD control group allowed for isolation of CUD-related associations in a population that often uses multiple substances. Using 16S rRNA gene sequencing, we examined alpha diversity, beta diversity, and taxa abundance between the groups and in relation to cannabis use patterns (frequency and amount; CUD group only), controlling for sequencing batch, age, sex, race, body mass index (BMI), and alcohol use.

**Results:**

Compared to the non-CUD SUD group, the CUD group exhibited significantly lower alpha diversity, distinct beta diversity, and differences in taxa abundance. Among those with CUD, greater cannabis use frequency was linked to lower diversity, while both frequency and amount were associated with higher abundances of strict anaerobes.

**Conclusions:**

CUD is associated with specific alterations in the oral microbiome, including lower diversity and taxonomic shifts. Associations with cannabis use patterns underscore the relevance of frequent and heavy use. Future research should explore the functional implications of these findings for CUD-related outcomes.

## 1. Introduction

Cannabis is the second most commonly used substance in young adults. The increasing legalization of cannabis for both medicinal and recreational purposes in the United States has led to a decline in the perceived risk of its use, especially among younger populations (Substance Abuse and Mental Health Services Administration SAMHSA, 2024). While cannabis offers therapeutic benefits for certain conditions, the change in perceived risk is concerning, as young adults are particularly vulnerable to the potential negative effects of cannabis (Fischer et al., 2020; Leinen et al., 2023). For example, earlier onset of cannabis use is associated with cognitive impairments, poor academic performance, and increased susceptibility to mental health disorders (Cyrus et al., 2021). Youth who use cannabis also face an increased risk of developing cannabis use disorder (CUD), which typically peaks during young adulthood (Lawn et al., 2022; Substance Abuse and Mental Health Services Administration SAMHSA, 2024). Moreover, the increasing potency of cannabis, driven by higher delta-9-tetrahydrocannabinol (THC) concentrations, further escalates this risk (Chandra et al., 2019; ElSohly et al., 2021; Freeman et al., 2021). It is important to note that current behavioral treatment options have limitations, and no medications for CUD have been approved by the U.S. Food and Drug Administration (FDA) (Askari et al., 2021; Sherman and McRae-Clark, 2016). Therefore, novel treatment options for CUD are urgently needed and will require a comprehensive knowledge of the physiological effects of cannabis.

Most of the research into novel pharmacological treatments for CUD has focused on the effects of cannabis on the brain, with comparatively little attention given to its interaction with peripheral systems like the microbiome. The human microbiome is a complex ecosystem of microorganisms that inhabit various parts of the body and play a crucial role in maintaining health (Ogunrinola et al., 2020). While the gut microbiome has attracted the most attention due to its large and diverse microbial community, the oral microbiome is increasingly recognized for its connections to brain function and behavior, making it a promising and underexplored target for novel therapeutics in conditions such as CUD. Disruptions in the oral microbiome have been linked to several neuropsychiatric and neurodegenerative conditions, with emerging evidence suggesting a role in substance use disorders (SUDs) (Kosciolek et al., 2021; Tao et al., 2024). Like the gut, the oral cavity communicates bidirectionally with the brain through neural, immune, and endocrine pathways (Adil et al., 2025; Bowland and Weyrich, 2022; Maitre et al., 2020; Narengaowa et al., 2021). Cranial nerves such as the trigeminal, olfactory, and vagus provide direct routes for oral bacteria and their byproducts to influence the central nervous system (Ha et al., 2023). Disruptions in the oral microbial community can also lead to increased permeability of the oral mucosa, allowing bacteria, bacterial products, and inflammatory mediators to enter systemic circulation (Gualtero et al., 2023). This may promote low-grade systemic inflammation, which is increasingly implicated in the pathophysiology of neuropsychiatric conditions, including SUDs (Kohno et al., 2019). Some of these circulating factors, such as pro-inflammatory cytokines, are capable of disrupting and crossing the blood-brain barrier, potentially triggering neuroinflammatory processes and altering neuronal function (Gryka-Marton et al., 2025). Additionally, oral bacteria can translocate to the gut, a process that has been observed at higher rates in conditions such as alcohol use disorder (AUD). This translocation may disrupt gut microbial composition, promote systemic inflammation, and influence brain function through gut-brain axis signaling (Ames et al., 2020; Kageyama et al., 2023; Schmidt et al., 2019). Taken together, these interconnected pathways highlight the potential of the oral microbiome as a novel target for therapeutic intervention in SUDs.

Few studies have specifically examined the relationship of the oral microbiome with cannabis use (Luo et al., 2021; Newman et al., 2019; Panee et al., 2024). This gap is notable given several factors that make cannabis particularly relevant to the oral microbial environment. Cannabis is almost exclusively consumed through the oral cavity, providing a direct route for it to alter the oral environment. Cannabis use has also been associated with oral health changes such as reduced salivary flow and lower oral pH, which create conditions that favor the growth of opportunistic pathogens and may contribute to microbial imbalance (Liu et al., 2020). To date, most research on cannabis and the oral microbiome has been limited to adult samples and has largely relied on comparisons between individuals that use cannabis and healthy controls. Our study addresses this gap by comparing individuals with CUD to those with other SUDs, allowing us to investigate cannabis-specific microbial alterations within the context of shared substance-related exposures. This distinction is important, as CUD often co-occurs with other SUDs (John et al., 2018). Furthermore, our focus on young adults aged 18–25 is based on both the developmental relevance of this period and the high prevalence of CUD in this population. Given that the oral microbiome continues to develop throughout young adulthood, this age range provides a critical window for identifying cannabis-related microbial alterations that may inform future efforts to improve prevention or treatment outcomes (Browning et al., 2024; McGrath et al., 2023). As such, the oral microbiome may be an important, albeit understudied, factor in mental health disorders such as CUD during young adulthood.

The goals of this study were to address two primary objectives: 1) To determine if the oral microbiome of young adults with CUD differ from those with other SUDs (non-CUD SUDs) so that CUD-specific alterations in the oral microbiome can be identified, and 2) To examine the association between the oral microbiome and the quantity of cannabis consumed and the frequency of its use in young adults with CUD. Through these objectives, we aim to identify oral microbiome patterns associated with CUD that may serve as a foundation for future research on microbial contributions to cannabis-related outcomes during young adulthood.

## 2. Methods and materials

### 2.1. Participants

Participants (N = 192) were aged 18–25 years and part of an overarching screening protocol for substance use studies (Davis et al., 2024). This study represents a secondary analysis of data collected during screening visits for the parent protocols. All participants in the CUD group (n = 129) met criteria for a CUD and had a positive urine drug screen for metabolites of THC. Diagnosis of another SUD was not exclusionary, as comorbidity with other SUDs is common among individuals with CUD (John et al., 2018). The non-CUD SUD group (n = 63) met criteria for a SUD other than cannabis. Having a non-CUD SUD control group allowed for isolation of CUD-related associations in a population that often uses multiple substances. Participants were excluded from the parent protocols if they were pregnant. For this specific study, participants were retrospectively excluded if they had used antibiotics, prebiotics, or probiotics within the past month or reported such use at the time of assessment, as the salivary microbiome is known to recover to baseline levels within that timeframe following these exposures (Lundtorp-Olsen et al., 2021; Zaura et al., 2015; Zaura et al., 2021). All participants provided written informed consent.

### 2.2. 16S rRNA gene amplification and sequencing

Participants provided a saliva specimen using a self-collection kit (OMNIgene•ORAL, OM-501 [DNA Genotek]) after verifying that they had not eaten, drunk, smoked, or chewed gum in the past 30 min. Samples were moved into −20 °C until analysis. Bacterial genomic DNA was extracted from the samples using established protocols at the Medical University of South Carolina (supplementary materials) and shipped for further processing and sequencing.

The V4 variable region of the 16S rRNA gene was amplified using PCR primers 515/806, with 30–35 cycles of amplification performed using the HotStarTaq Plus Master Mix Kit (Qiagen, USA). PCR conditions included initial denaturation at 95°C for 5 min, followed by cycles of denaturation at 95°C for 30 s, annealing at 53°C for 40 s, extension at 72°C for 1 min, and a final extension step at 72°C for 10 min. Post-amplification, PCR products were assessed using 2 % agarose gel electrophoresis to confirm successful amplification and determine relative band intensities. Samples were multiplexed with unique dual indices and pooled based on molecular weight and DNA concentrations. Pooled samples underwent purification using calibrated Ampure XP beads. Subsequently, the purified PCR product was used to prepare an Illumina DNA library, followed by sequencing on a MiSeq platform at MR DNA (www.mrdnalab.com, Shallowater, TX, USA), following manufacturer guidelines.

### 2.3. Upstream sequence analysis of microbiome data

The DADA2 pipeline was used to check quality and process reads (version 1.3). First, we filtered, trimmed, and merged reads for the four batches separately. Primers and adapters were cut from the reads using trim left (50, 50); forward and reverse reads were truncated at 220 bp and 200 bp, respectively; and low-quality reads were removed using the DADA2 default filtering parameters. We then pooled the amplicon sequence variant (ASV) tables and removed chimeras. Finally, we assigned the taxonomy using the Human Oral Microbiome Database (Chen et al., 2010). The distribution of sequencing depth and rarefaction curve are presented in Figures S1–S2.

### 2.4. Assessments

Questionnaires were used to capture participants’ demographic and background information, medication use, physical health, mental health, and substance use history. Participants were administered a modified Timeline Followback (TLFB) to determine how much cannabis and other substances they consumed and at what frequency and amount (Sobell and Sobell, 1992). Participants were asked to report the types of cannabis they used, and for each type to estimate the amount they typically used in grams or milligrams. Then they were asked for each prior day to report the types used and the corresponding number of units. For each type, units were multiplied by estimated grams for that type, and grams used in the 7 days prior to saliva collection was summed.

The Mini International Neuropsychiatric Interview (MINI) was administered to diagnose substance use and other psychiatric disorders in participants (Sheehan et al., 1998). The MINI is a brief, structured diagnostic tool that has been updated to assess Diagnostic and Statistical Manual for Mental Disorders-5 (DSM-5) criteria (“Diagnostic and statistical manual of mental disorders: DSM-5^™^, 5th ed,” 2013). The MINI does not assess nicotine dependence; therefore, we used “suggestive nicotine dependence,” which is defined as a score of six or above on the Modified Version of the Fagerstrom Tolerance Questionnaire (mFTQ) and four or above on the Penn State Electronic Cigarette Dependence Index (PSECDI) (Foulds et al., 2015; Prokhorov et al., 2000; Prokhorov et al., 1996).

### 2.5. Statistical analysis

Statistical analyses were conducted in R Software (R Core Team, 2023). Plots were generated with the ggplot2 and phyloseq packages (McMurdie and Holmes, 2013; Wickham, 2016). For objective 1, inverse probability weights (IPWs) were used to mitigate the potential impact of confounding on our results, given large differences in certain covariates between groups (Rosenbaum and Rubin, 1983). IPWs represent the inverse probability of participants being in their observed exposure group – here, having CUD or not– given how their covariate profile compares to what is typical for that group. Thus, the more atypical the covariate values for participants’ exposure status, the greater weight their data is given. This reweighting of the sample should balance confounding characteristics between the two groups. Standardized mean differences between groups were calculated for all covariates (sequencing batch, age, sex, race, BMI, and drinks over the past 7 days) before and after weighting to assess how well weighting achieved balance between groups (a standardized difference <10 % is the accepted rule of thumb) (Austin and Stuart, 2015). Love plots visualizing these differences can be found in the supplementary materials (Figures S3–S4).

Subsequent regressions were both IPW-weighted and adjusted for the same set of covariates used to generate the weights, resulting in a doubly robust analysis that provides two opportunities to nullify the influence of confounding variables on the exposure–outcome relationship. Propensity scores were also included as covariates in the models to further account for potential confounding. Standard errors/confidence intervals were adjusted to incorporate the uncertainty introduced by IPWs. The WeightIt package was used for the analyses (Greifer, 2025).

For objective 2, an outlier cutoff of greater than 3 z-scores was applied to the cannabis grams data, leading to the exclusion of three participants (Figures S5–S6). All analyses in objective 2 included sex-at-birth, drinks over the past 7 days, body mass index, sequencing batch, age, and race as covariates.

#### 2.5.1. Alpha diversity

Alpha diversity refers to the variety and abundance of taxa within an environment. In this study, we calculated several alpha diversity indices, including Shannon, Simpson, Pielou’s evenness, and Chao1 using the microbiome package (Shetty, 2012–2019). These indices help to quantify the richness (number of different taxa) and evenness (distribution of those taxa) of the microbial community. Linear regression models were performed using the lm() function from the stats package in R to assess the association of each alpha diversity index. Significance for alpha diversity analyses was set at *p* < 0.05.

#### 2.5.2. Beta diversity

Beta diversity evaluates the differences in taxonomic composition between groups. We assessed it using three dissimilarity metrics: Bray-Curtis, which considers taxa abundance; Jaccard, which focuses on taxa presence and absence; and Aitchison, which addresses the compositional nature of the data. These calculations were performed using the phyloseq package and visualized using Principal Coordinate Analysis (PCoA) plots. Differences in bacterial community profiles between groups were statistically examined using Permutational Multivariate Analysis of Variance (Anderson, 2017; Oksanen et al., 2024). Significance for beta diversity analyses was set at *p* < 0.05.

#### 2.5.3. Differential abundance

Differential abundance refers to the identification of specific taxa that vary in abundance between the groups. For differential abundance analyses, taxa with a prevalence of less than 10 % were excluded. Microbial abundance differences were identified using the Microbiome Multivariable Association with Linear Models (MaAslin2) package using centered log-ratio transformation (CLR), which accounts for the compositional nature of microbiome data (Mallick et al., 2021). Unlike alpha and beta diversity, which summarize microbial communities using single values per sample, differential abundance analyzes each taxon individually to identify specific taxa that vary significantly between groups. To account for the increased likelihood of false positives resulting from this multiple testing, the Benjamini-Hochberg procedure was applied, with a default *q*-value threshold of 0.25 used to define significance.

All analyses were performed at the genus and species levels. Feature tables are provided in the supplementary materials to support meta-analyses and future study power calculations.

## 3. Results

### 3.1. Participant characteristics

Detailed sociodemographic and clinical variables are presented in Table 1. The study included 192 participants, with 129 in the CUD group and 63 in the non-CUD SUD group. Most participants were female (67.44 % in CUD and 58.73 % in non-CUD SUD) and White (70.54 % in CUD and 90.48 % in non-CUD SUD). Within the CUD group, severity levels of CUD varied from mild (n = 22, 17.05 %) to moderate (n = 33, 25.58 %) to severe (n = 74, 57.36 %). In addition to meeting CUD criteria, some participants in the CUD group also met criteria for suggestive nicotine dependence (n = 65, 50.39 %), alcohol use disorder (AUD; n = 69, 53.49 %), stimulant use disorder (n = 9, 6.98 %), cocaine use disorder (n = 12, 9.30 %), opioid use disorder (n = 2, 1.55 %), hallucinogen disorder (n = 11, 8.53 %), inhalant use disorder (n = 3, 2.33 %), and sedative use disorder (n = 1, 0.78 %). Within the CUD group, 36 (27.91 %) participants met criteria for CUD only, 28 (21.71 %) met criteria for both CUD and AUD, 24 (18.60 %) for CUD and suggestive nicotine dependence, and 41 (31.78 %) met criteria for all three (CUD, AUD, and suggestive nicotine dependence). In the non-CUD SUD group, participants met criteria for suggestive nicotine dependence (n = 31, 49.21 %), AUD (n = 53, 84.13 %), cocaine use disorder (n = 2, 3.17 %), and hallucinogen use disorder (n = 1, 1.59 %). Of the non-CUD SUD participants, 32 (50.80 %) participants met criteria for AUD only, 10 (15.87 %) for suggestive nicotine dependence only, and 21 (33.33 %) for both AUD and nicotine dependence. Among the participants, 16 from the non-CUD SUD group reported cannabis use in the past 7 days, whereas nearly all participants in the CUD group (except for 2) reported using cannabis during this period. Leaf/bud material was reported by both groups as their main cannabis use method (CUD: 70.54 %, non-CUD SUD: 20.63 %), indicating that most participants smoked their cannabis. The number of different administration methods reported ranged from 0 to 6, and only 5 participants reported using a single method. This limited our ability to isolate the effects of any one route of administration.

### 3.2. Microbiome

We generated a total of 9296 amplicon sequence variants (ASVs) from 616,930,124 high-quality, nonchimeric reads. The ASVs were classified into 183 genera and 616 species.

#### 3.2.1. Aim 1: microbial diversity and abundance in CUD vs. Non-CUD SUD

##### 3.2.1.1. Diversity.

**Fig. 1.**
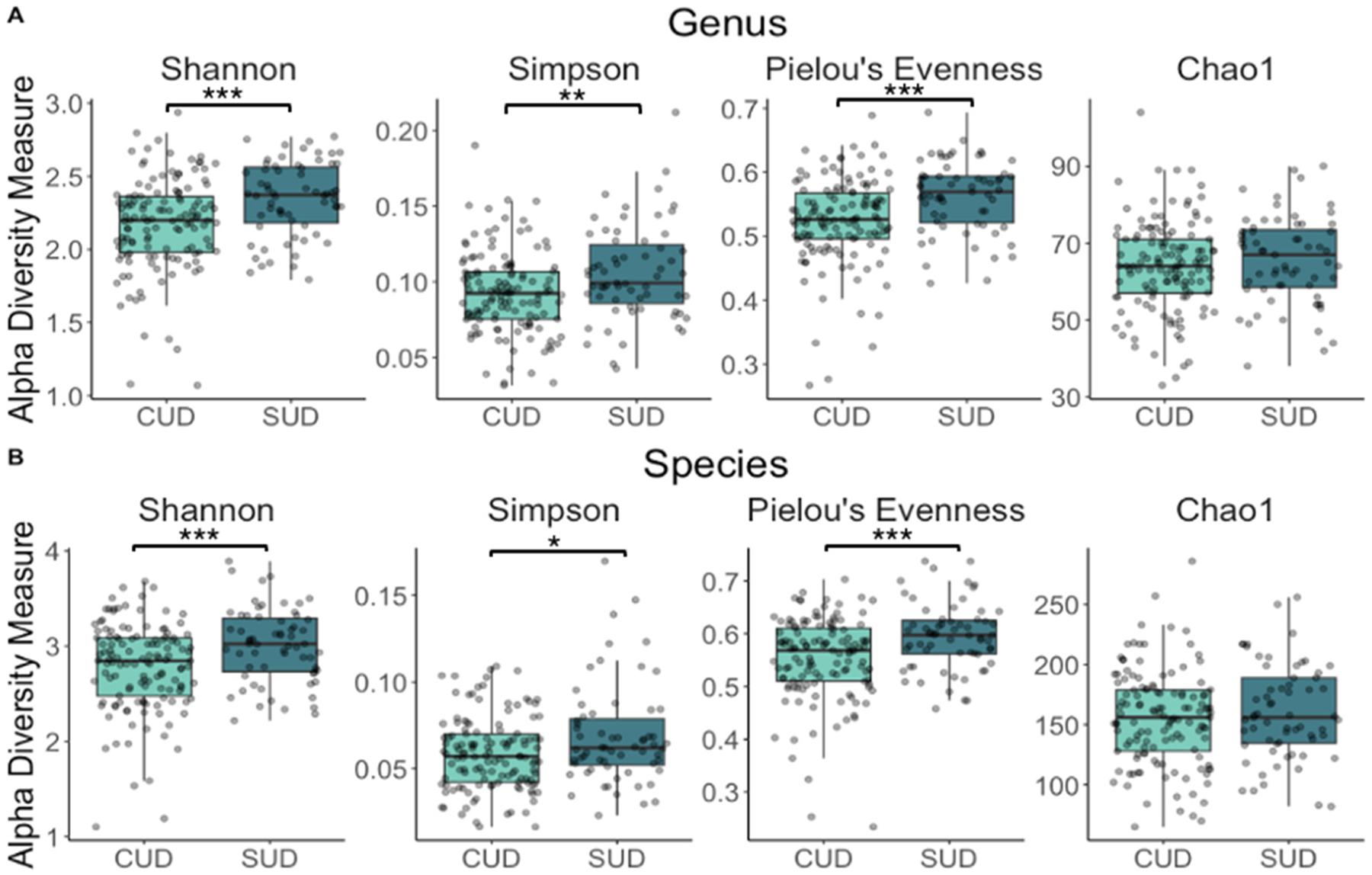
Alpha diversity in the cannabis use disorder (CUD) and non-CUD substance use disorder (SUD) groups. A) The CUD group had significantly lower Shannon (*p* < 0.001), Simpson (*p* = 0.004), and Pielou’s Evenness (*p* < 0.001) scores at the genus level. B) The CUD group had significantly lower Shannon (*p* < 0.001), Simpson (*p* = 0.010), and Pielou’s evenness (*p* < 0.001) scores at the species level.

At the genus level, alpha diversity was significantly lower in the CUD group as indicated by the Shannon index (b= −0.198, 95 % CI= [−0.282, −0.114], *p* = <0.001), the Simpson index (b= −0.014, 95 % CI= [−0.023, −0.005], *p* = 0.004), and the Pielou’s evenness index (b= −0.042, 95 % CI= [−0.060, −0.024], *p* = <0.001) (Fig. 1A, Supplementary Table 1). At the species level, alpha diversity was also significantly lower in the CUD group using the Shannon (b= −0.254, 95 % CI= [−0.379, −0.129], *p* = <0.001), Simpson (b= −0.009, 95 % CI= [−0.016, −0.002], *p* = 0.010) and Pielou’s evenness (b= −0.043, 95 % CI= [−0.063, −0.023], *p* = <0.001) indices (Fig. 1B, Table S1).

**Fig. 2.**
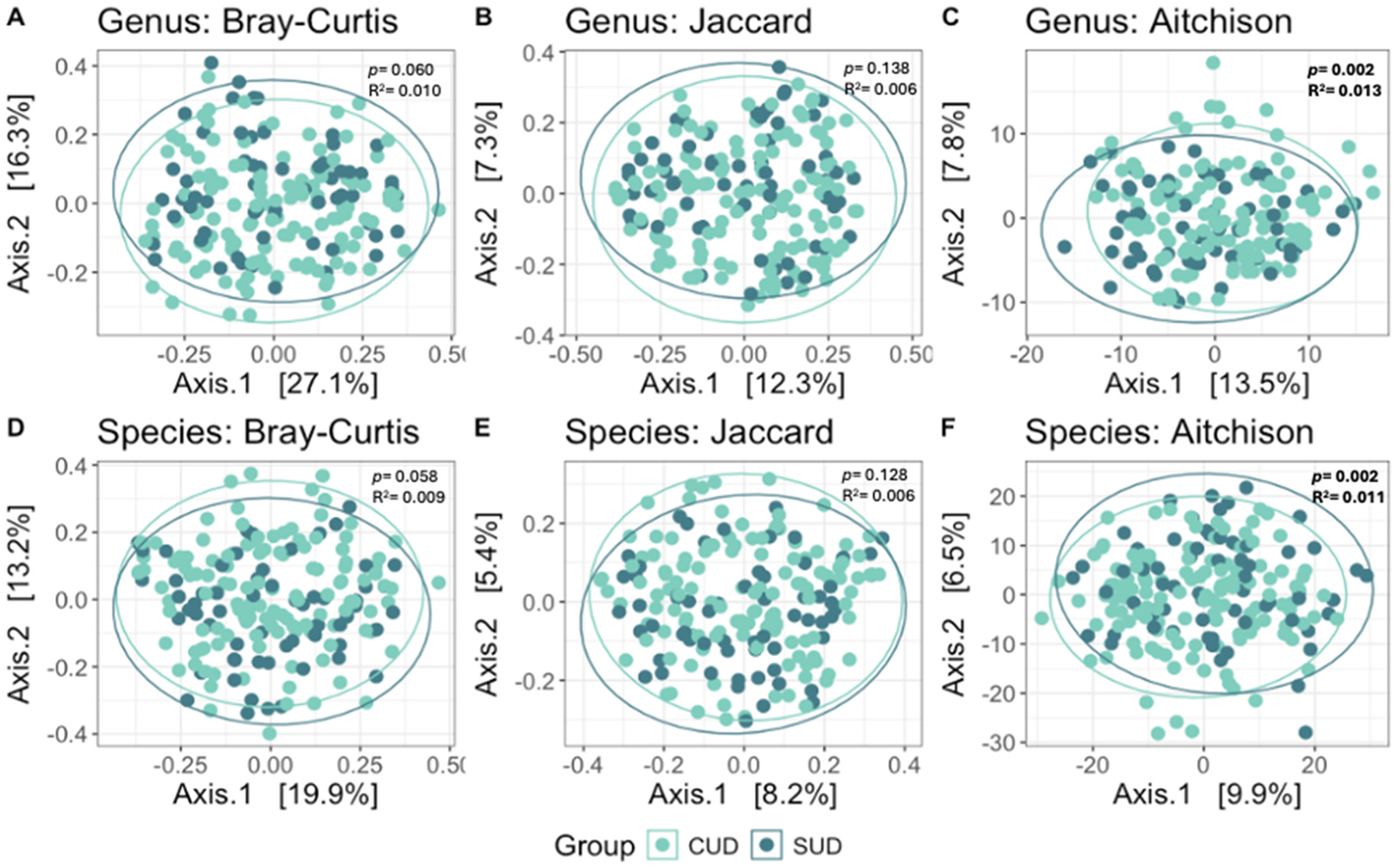
Beta Diversity in the Cannabis Use Disorder (CUD) and non-CUD Substance Use Disorder (SUD) groups. A-C) Genus-level principal coordinates analysis (PCoA) plots using the Bray-Curtis, Jaccard, and Aitchison dissimilarity metrics. D-F) Species-level PCoA plots using the Bray-Curtis, Jaccard, and Aitchison dissimilarity metrics.

Beta diversity analyses revealed significant differences between the CUD and non-CUD SUD groups as assessed by the Aitchison dissimilarity metric at both the genus (*p* = 0.002, R^2^= 0.013) and species (*p* = 0.001, R^2^= 0.011) levels (Fig. 2, Table S2).

##### 3.2.1.2. Abundance.

**Fig. 3.**
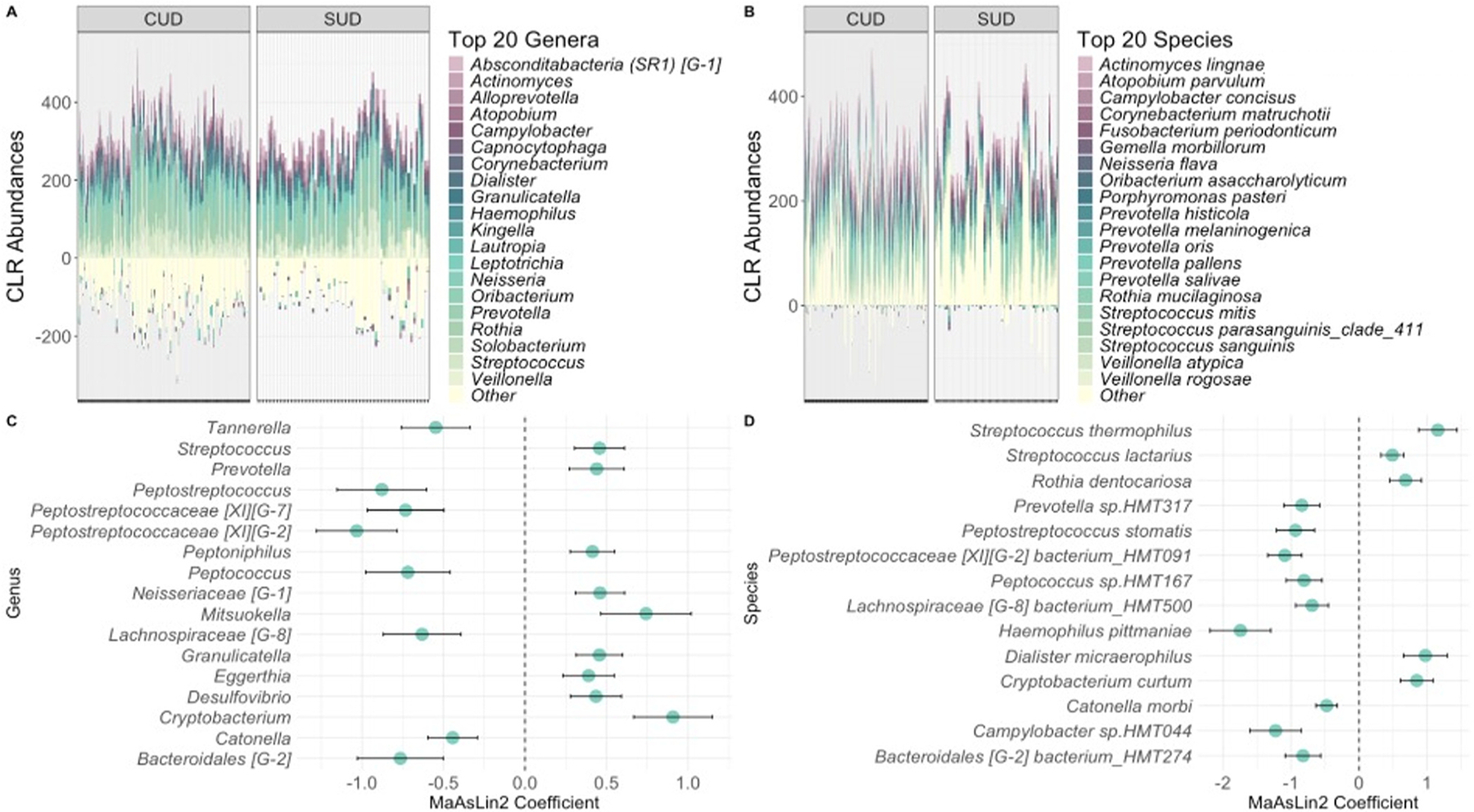
Centered log ratio (CLR) abundances in the Cannabis Use Disorder (CUD) and non-CUD Substance Use Disorder (SUD) groups at the species and genus levels. A) Top 20 genera abundances by substance group. B) Top 20 species abundances by substance group. C) Genera that were significantly different between the CUD and non-CUD SUD groups, D) Species that were significantly different between the CUD and non-CUD SUD groups. All q-values < 0.025.

Abundance profiles of the top 20 genera and species for each group are presented in Fig. 3. Seventeen genera differed significantly in abundance between the CUD and non-CUD SUD groups (Fig. 3C). Of these, eight were less abundant in the CUD group: *Peptostreptococcaceae [XI][G-2]* (coef= −1.033, *q*= 0.012), Peptostreptococcus (coef= −0.879, *q*= 0.127), *Bacteroidales [G-2]* (coef= −0.765, *q*= 0.138), *Peptostreptococcaceae [XI][G-7]* (coef= −0.733, *q*= 0.127), *Peptococcus* (coef= −0.719, *q*= 0.171), *Lachnospiraceae [G-8]* (coef= −0.632, *q*= 0.196), *Tannerella* (coef= −0.548, *q*= 0.196), and *Catonella* (coef= −0.444, *q*= 0.138). Genera that were more abundant in the CUD group included *Eggerthia* (coef= 0.390, *q*= 0.238), *Peptoniphilus* (coef= 0.414, *q*= 0.127), *Desulfovibrio* (coef= 0.435, *q*= 0.171), *Prevotella* (coef= 0.440, *q*= 0.196), *Granulicatella* (coef= 0.456, *q*= 0.127), *Streptococcus* (coef= 0.457, *q*= 0.129), *Neisseriaceae [G-1]* (coef= 0.460, *q*= 0.127), *Mitsuokella* (coef= 0.742, *q*= 0.196), and *Cryptobacterium* (coef= 0.908, *q*= 0.043).

We also identified fourteen species that were significantly different between the groups (Fig. 3D). The nine species that were less abundant in the CUD group included *Haemophilus pittmaniae* (coef= −1.746, *q*= 0.052), *Campylobacter sp.HMT044* (coef= −1.228, *q*= 0.162), *Peptostreptococcaceae [XI][G-2] bacterium_HMT091* (coef= −1.093, *q*= 0.018), *Peptostreptococcus stomatis* (coef= −0.935, *q*= 0.150), *Prevotella sp. HMT317* (coef= −0.843, *q*= 0.168), *Bacteroidales [G-2] bacterium_HMT274* (coef= −0.826, *q*= 0.175), *Peptococcus sp.HMT167* (coef= −0.809, *q*= 0.177), *Lachnospiraceae [G-8] bacterium_HMT500* (coef= −0.692, *q*= 0.218), and *Catonella morbi* (coef= −0.477, *q*= 0.180). The species with higher abundances consisted of *Streptococcus lactarius* (coef= 0.489, *q*= 0.203), *Rothia dentocariosa* (coef= 0.683, *q*= 0.203), *Cryptobacterium curtum* (coef= 0.848, *q*= 0.146), *Dialister micraerophilus* (coef= 0.975, *q*= 0.183), and *Streptococcus thermophilus* (coef= 1.158, *q*= 0.034).

#### 3.2.2. Aim 2: microbial diversity and abundance differences associated with quantity of cannabis consumed and frequency of cannabis use (n = 126, CUD group only)

##### 3.2.2.1. Patterns of use and diversity.

In the CUD group, the number of cannabis use days was negatively correlated with microbial diversity at the genus (b= −1.432, 95 % CI= [−2.564, −0.300], *p* = 0.014) and species (b= −4.848, 95 % CI= [−8.706, −0.991], *p* = 0.014) levels using the Chao1 index. No significant associations were observed between the number of cannabis grams or grams per use day with any alpha diversity indices (Table S3).

The number of cannabis use days was also significantly associated with the composition of the microbial community as measured by Aitchison dissimilarity at the species level (R^2^= 0.012, *p* = 0.001; Table S4).

##### 3.2.2.2. Patterns of use and abundance.

**Fig. 4.**
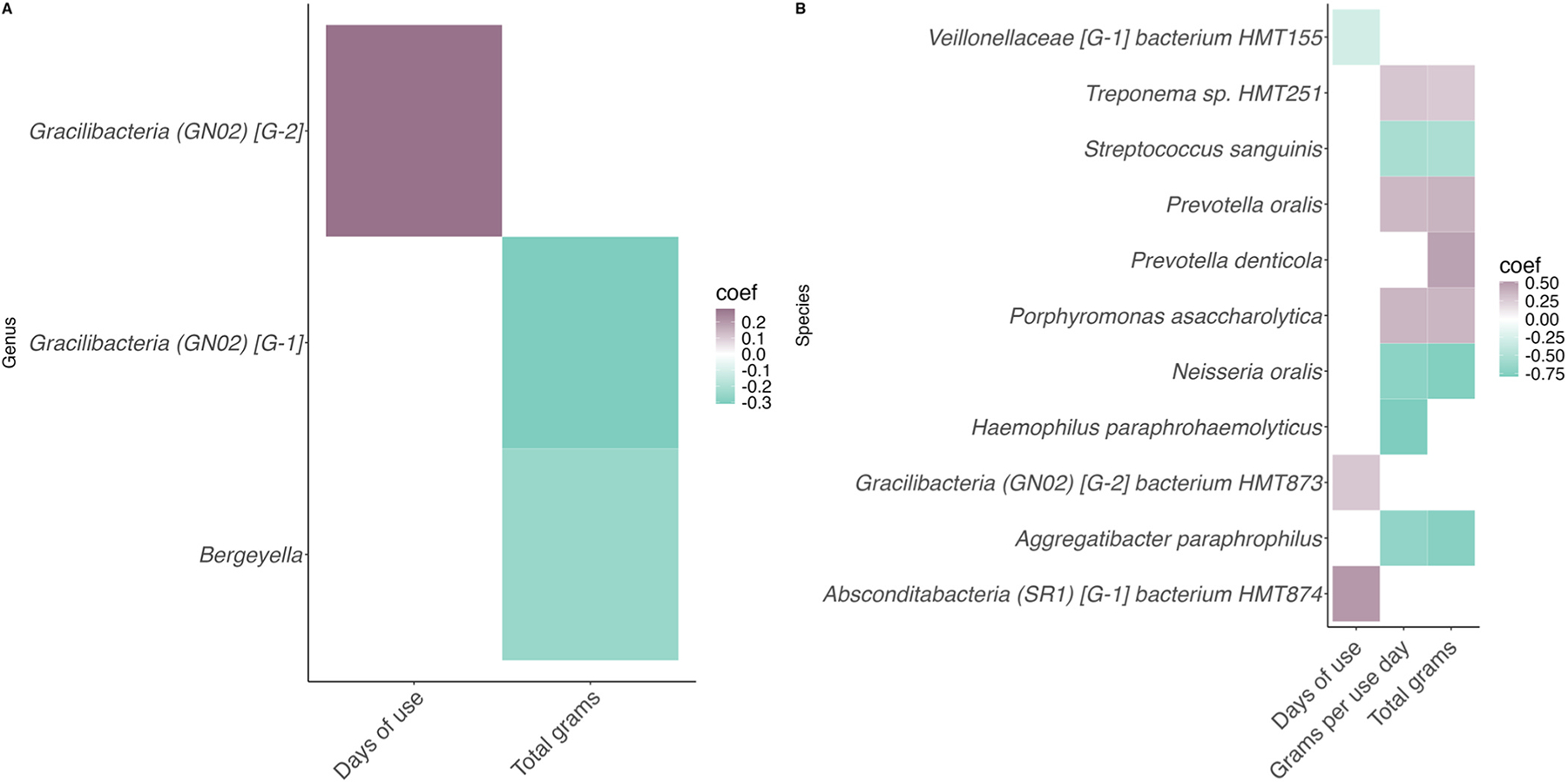
Taxa significantly associated with quantity of cannabis consumed and frequency of cannabis use among young adults with CUD (n = 126). A) Significant associations at the genus level, B) Significant associations at the species level. All q-values < 0.025.

Taxa signficantly associated with the quantity of cannabis consumed and frequency of cannabis use in participants with CUD are presented in Fig. 4.

The number of cannabis use days was positively correlated with abundances of the genus *Gracilibacteria [GN02] [G-2]* (coef = 0.281, *q*= 0.113) as well as the species *Gracilibacteria (GN02) [G-2] bacterium HMT873* (coef= 0.274, *q*= 0.209) and *Absconditabacteria (SR1) [G-1] bacterium HMT874* (coef= 0.513, *q*= 0.209). Only one species, *Veillonellaceae [G-1] bacterium HMT155* (coef= −0.296, *q*= 0.159), was negatively correlated.

The total number of cannabis grams over the past 7 days was negatively correlated with the abundances of two genera: *Gracilibacteria [GN02] [G-1]* (coef= −0.310, *q*= 0.231) and *Bergeyella* (coef= −0.252, *q*= 0.232). In addition, abundances of the species *Porphyronomas asaccharolytica* (coef= 0.362, *q*= 0.134), *Prevotella denticola* (coef= 0.467, *q*= 0.202), *Prevotella oralis* (coef= 0.380, *q*= 0.113), and *Treponema sp. HMT251* (coef= 0.268, *q*= 0.227) were positively correlated with the total number of cannabis grams. *Aggregatibacter paraphrophilus* (coef= −0.745, *q*= 0.157), *Neisseria oralis* (coef= −0.763, *q*= 0.110), and *Streptococcus sanguinis* (coef= −0.524, *q*= 0.211) were negatively correlated.

Finally, the number of grams per use day was not associated with any genera but was positively correlated with abundances of the species *Porphyromonas asaccharolytica* (coef= 0.367, *q*= 0.136) and *Prevotella oralis* (coef= 0.355, *q*= 0.161), *Treponema HMT251* (coef= 0.283, *q*= 0.198) and was negatively correlated with abundances of *Streptococcus sanguinis* (coef= −0.541, *q*= 0.189), *Haemophilus paraphrohaemolyticus* (coef= −0.793, *q*= 0.219), and *Aggregatibacter paraphrophilus* (coef= −0.678, *q*= 0.226).

## 4. Discussion

In this study, we aimed to 1) determine if the oral microbiome of young adults with CUD differs from those with non-CUD SUDs, and 2) determine whether differences in the oral microbiome in the CUD group are associated with the quantity of cannabis consumed and/or the frequency of its use. Identifying microbiome differences specific to CUD, as opposed to other SUDs, is crucial for treatment development given the high prevalence of comorbid SUDs among young adults with CUD. In our study, after we robustly accounted for the potential impacts of confounding factors, the CUD group exhibited significantly lower alpha diversity, distinct beta diversity, and differences in the abundances of several taxa compared to the non-CUD SUD group. Additionally, within the CUD group, both the quantity of cannabis consumed, and the frequency of its use were associated with differences in microbiome diversity and taxa abundances. These findings point to a potential relationship between the oral microbiome and CUD that could have important implications for oral health and could provide new avenues for developing CUD-specific therapeutics.

Our analysis revealed that alpha diversity was significantly lower in the CUD group, suggesting a less diverse oral microbiome compared to the non-CUD SUD group. Specifically, significantly lower diversity scores were observed in the CUD group using the Shannon, Simpson, and Pielou’s evenness indices at the genus level. At the species level, scores on the Shannon and Pielou’s evenness indices were significantly lower in the CUD group. While the Simpson and Shannon indices capture both the richness and evenness of taxa, Pielou’s evenness index focuses solely on evenness, suggesting that lower evenness (i.e., lower distribution of taxa) may be a key factor in the observed lower diversity in the CUD group.

The frequency of cannabis use appears to be more closely associated with changes in richness (i.e., the number of taxa), as evidenced by the negative correlation between the number of cannabis use days and Chao1, an index that specifically measures richness, at both the genus and species levels in the CUD group. This finding suggests that richness may be more sensitive to changes in cannabis use over a short period, such as 7 days. In contrast, differences in evenness may be more obvious when comparing groups, as it reflects the distribution of species rather than their abundance or presence, making it less responsive to short-term fluctuations in cannabis use. Overall, these findings suggest that cannabis use is associated with lower microbial diversity, both at the group level, differentiating young adults with CUD from those with non-CUD SUD, and in relation to cannabis use frequency.

The lower diversity associated with cannabis use can have several possible explanations. One possibility is that cannabis has an antimicrobial effect, attributed to cannabidiol’s ability to disrupt bacterial cell membranes and inhibit the production of biofilms (Blaskovich et al., 2021). Another possible explanation is that the heat from smoking cannabis, the most common method of use in our cohort, kills microbes by denaturing their proteins and disrupting their cell membranes (Cebrián et al., 2017). Finally, the reduced salivary flow associated with cannabis use may make it more difficult for beneficial microbes to survive and maintain a balanced microbial community (Hayashi et al., 2015). While cannabis use may lead to lower microbial diversity through antimicrobial effects, heat exposure, or reduced salivary flow, it is also possible that pre-existing alterations in the oral microbiome contribute to cannabis use. Individuals with SUDs who exhibit lower microbial diversity have been found to report greater negative reinforcement experiences related to their primary substance of use (Kosciolek et al., 2021). This suggests that low oral microbiome diversity may contribute to patterns of cannabis use driven by the desire to avoid negative feelings or withdrawal symptoms, though more research is needed to fully understand this connection.

Beta diversity analyses revealed significant differences between the groups at both the genus and species levels when assessed using the Aitchison dissimilarity metric, which accounts for differences in both taxa abundance and presence/absence. Additionally, the number of cannabis use days was significantly associated with beta diversity, as measured by Aitchison dissimilarity at the species level. However, no significant differences were found when using the Jaccard index, which only considers species presence or absence, or the Bray-Curtis index, which primarily measures species abundance. These findings suggest that cannabis-related alterations in the oral microbiome may involve more complex, community-wide shifts that are not limited to the presence or abundance of individual taxa. Such patterns may be especially relevant in early or chronic stages of cannabis use, where disruption of microbial community structure could occur in complex ways.

Taken together, the significant differences in both alpha and beta diversity suggest that the oral microbiome of the CUD group has a distinct composition, marked by a lower number of taxa that are unevenly distributed compared to other SUDs. These findings are consistent with a study reporting lower alpha and distinct beta diversity in adults with CUD compared to controls (Luo et al., 2021), but differ from another study that found no diversity differences between individuals with chronic cannabis use and controls in the salivary microbiome (Panee et al., 2024). While these differences may stem from age or other sample-related factors, they could also suggest that changes to diversity are specific to CUD rather than cannabis use alone, but more research is needed to clarify this relationship.

Interpreting these diversity differences in the context of CUD is challenging, given that the role of oral microbial diversity is not as well-defined as in other microbial environments like the gut. Both lower and higher microbial diversity have been associated with negative health outcomes in different neuropsychiatric and neurodegenerative conditions, making it difficult to define a “healthy” level of diversity. However, the presence of diversity differences, regardless of direction, suggests differences in the microbial community that may have biological relevance. In this context, the lower diversity observed in the CUD group may reflect a microbial profile that is characteristic of CUD rather than inherently better or worse than that of individuals with other SUDs. Follow-up studies incorporating microbial function or host response will be critical for clarifying the significance of these findings and their potential relevance to cannabis-related health outcomes.

When investigating differences in specific taxa between the groups, we identified seventeen genera and fourteen species that were differentially abundant between young adults with CUD and young adults with non-CUD SUD. We observed higher abundances of the genera *Streptococcus* and *Prevotella* in the CUD group, which aligns with findings from gut microbiome studies in adults with CUD and adults with chronic cannabis use, respectively (Luo et al., 2021; Panee et al., 2018). However, those studies also identified higher abundances of the species *Megasphaera micronuciformis* and *Viellonella atypica* with cannabis use, which we did not observe in our study. These findings suggest that cannabis use may lead to broad changes in the microbiome, such as shifts at the genus level, but that specific changes related to CUD may be more pronounced at the species level.

In addition, we identified several taxa associated with both the quantity of cannabis consumed and the frequency of its use among participants with CUD. Specifically, the number of cannabis use days was linked to one genus and three species, the total amount of cannabis consumed over the past 7 days was associated with two genera and three species, and the number of grams consumed per use day was associated with five species. Interestingly, all taxa that were positively correlated with more cannabis use were either strict anaerobes or likely to be strict anaerobes, meaning they cannot survive in the presence of oxygen. This finding aligns with previous research that shows smoking cigarettes makes the oral cavity more anaerobic (Mohammed et al., 2024; Wu et al., 2016), suggesting that higher cannabis use among young adults with CUD may contribute to a shift in the oral microbiome toward a more anaerobic composition, potentially increasing the risk for oral health issues associated with cannabis use, such as periodontal disease and other oral infections (Thomson et al., 2008).

Two measures of cannabis consumption, the total grams consumed in the past week and the grams consumed per use day, were associated with several of the same species of microbes. These included *Aggregatibacter paraphrophilus*, *Streptococcus sanguinis*, and *Neisseria oralis*, all of which exhibited lower abundances with higher cannabis consumption. Our finding that *Streptococcus sanguinis* was negatively correlated with cannabis use appears to be in conflict with previous research, which observed higher abundances of this species in individuals with chronic cannabis use compared to nonsmoking control participants (Panee et al., 2024). However, it has also been reported that the non-psychoactive phytocannabinoid Cannabigerol (CBG) has antibacterial effects against *Streptococcus sanguinis* (formerly *Streptococcus sanguis*), suggesting that the specific composition of cannabis may play a role in these differing results, along with the other factors mentioned above (Aqawi et al., 2021). Our study did not capture fine-grained details on the cannabinoid content of the cannabis used by participants, underscoring the need for future research in this area.

Both the total number of cannabis grams over the past seven days as well as the amount of cannabis per use day were positively correlated with abundances of *Porphyromonas asaccharolytica*, *Prevotella oralis*, and *Treponema sp. HMT251*. Interestingly, studies have reported finding several *Treponema* species in various regions of postmortem brains from individuals with Alzheimer’s disease and control participants, including the trigeminal ganglia, pons, hippocampus, and frontal lobe cortex (Riviere et al., 2002; Siddiqui et al., 2019). However, it is important to note that the specific species identified in this study, *Treponema sp. HMT251*, has been little studied, and more research is needed to determine its potential association with the brain. In addition to the shared species, abundances of *Prevotella denticola* were positively correlated with the total number of grams, and *Haemophilus paraphrohaemolyticus* was negatively associated with the number of grams per use day. These findings suggest that, while the microbial shifts observed in relation to cannabis use are generally similar across different measures of consumption, the frequency and intensity of cannabis use may play distinct roles in shaping the oral microbiome.

While our findings shed light on the potential impact of CUD and cannabis use on the oral microbiome, several limitations must be acknowledged. First, the cross-sectional design limits causal inference, and longitudinal research is needed to determine whether observed microbial alterations are a cause or consequence of cannabis use. Second, because we relied on secondary data from existing substance use protocols, we were unable to design a demographically balanced sample, and the overall sample was predominantly white and female. While this limits generalizability, our study differs from previous substance use research, which has historically overrepresented White men (Gunn et al., 2022). Third, the lack of a non-SUD comparator group may be perceived as a limitation; however, our approach fills a gap in the literature as, to our knowledge, all the previous studies have focused on comparisons between individuals with SUDs and control participants with no SUDs. Our findings offer novel insights and reflect the comorbidity commonly observed in substance-using populations and help isolate the CUD-specific alterations in individuals who use cannabis. We explored the distribution of other substance use disorders within the sample to determine whether alcohol- and nicotine-related differences could be disentangled; however, the subgroup sizes were not large enough to support adequately powered comparisons. *Future studies with larger, more stratified samples will be necessary to disentangle the independent and interactive effects of co-occurring SUDs on the oral microbiome*. Moreover, we attempted to mitigate demographic imbalances and potential confounding through rigorous statistical approaches, including both covariate adjustment and propensity score matching on age, sex, race, and alcohol use. Nonetheless, residual confounding remains a possibility. Fourth, we did not assess dietary intake or oral health status, both of which can influence the oral microbiome. However, our sample consisted of generally healthy young adults (ages 18–25), who are less likely to experience significant oral pathology or major dietary fluctuations compared to older or clinical populations. While not a direct measure of diet, we included BMI as a covariate to approximate general nutritional status, though we recognize it does not capture specific dietary patterns or nutrient intake. Although this remains a common limitation in microbiome research due to the complexity and lack of standardized assessments, future studies should prioritize the inclusion of objective, validated measures of both oral health and diet to better account for these potential confounders. Similarly, objective measures for cannabis use quantity and frequency are currently lacking. As such, we relied on self-reported data, which is standard in the field but may introduce recall bias(Baker et al., 2018). Finally, our microbial profiling relied on 16S rRNA sequencing, which enables broad taxonomic comparisons but lacks species-level resolution and does not capture microbial function. Future work using shotgun metagenomics or integrated multi-omic approaches will be critical for identifying species-specific and functional differences that may underlie the observed associations. Despite these limitations, this study provides important preliminary insights into cannabis-related oral microbiome alterations and highlights key directions for future research aimed at clarifying their clinical relevance.

In conclusion, our study suggests that the oral microbiome of young adults with CUD exhibits distinct patterns compared to those with other SUDs. These findings extend prior research that has identified differences between individuals with CUD and healthy controls and highlight the importance of future research aimed at determining whether these microbial differences are unique to CUD and clinically meaningful. If future longitudinal and mechanistic studies confirm that these microbial patterns are not only consequences but also contributors to the maintenance of cannabis use, then interventions aimed at restoring microbial diversity such as probiotics, prebiotics, or oral hygiene strategies may play a supportive role in improving treatment outcomes. This work represents an early but important step in disentangling the complex biological factors that may contribute to CUD and identifying new directions for intervention.

## Supplementary Material

Appendix A. Supplementary material

## Figures and Tables

**Table 1:** Participant characteristics.

	CUD (n=129)	Non-CUD SUD (n=63)	Group comparison
**Age, y, mean (SD)**	21.16 (1.89)	21.47 (1.88)	*p=* 0.230
**Sex, Female, No. (%)**	87 (67.44)	37 (58.73)	*p=* 0.306
**Race, No. (%)**			
White	91 (70.54)	57 (90.48)	*p=* 0.004
Non-white	38 (29.46)	6 (9.52)	
**Ethnicity, Hispanic/Latinx, No. (%)**	15 (10.79)	6 (9.52)	*p=* 0.848
**BMI, kg/m** ^**2**^ **, mean (SD)**	24.45 (6.08)	24.51 (5.65)	*p=* 0.634
**Cannabis use**			
**Cannabis use disorder severity, No. (%)** ^a^			
Mild	22 (17.05)	-	
Moderate	33 (25.58)	-	
Severe	74 (57.36)	-	
**Cannabis use days, mean (SD)** ^b^	5.98 (1.64)	0.92 (2.10)	*p* < 0.001
**Cannabis grams per use day, mean (SD)** ^b^	1.91 (3.26)	0.26 (0.98)	*p* < 0.001
**Cannabis grams past 7 days, mean (SD)** ^b^	12.32 (22.47)	1.53 (6.72)	*p* < 0.001
**Primary cannabis use method, No. (%)**			
Edibles/drink/tinctures	15 (11.63)	1 (6.25)	*p* < 0.001
Leaf/bud material	91 (70.54)	13 (81.25)	
Wax/dabs/concentrates/oils	18 (13.95)	2 (12.5)	
Other	5 (3.88)	-	
**Number of cannabis use methods over the past 7 days, No. (%)**			*p* < 0.001
0	2 (1.55)	47 (74.60)	
1	36 (27.91)	7 (11.11)	
2	50 (38.76)	7 (11.11)	
3	22 (17.05)	2 (3.18)	
4	13 (10.08)	-	
5	4 (3.10)	-	
6	2 (1.56)	-	
**Other substance use** ^b^			
**Alcohol**			
Alcohol use days, mean (SD)	1.50 (1.55)	2.30 (1.66)	*p* < 0.001
Total number of drinks, mean (SD)	6.16 (8.72)	11.58 (10.76)	*p* < 0.001
**Nicotine**			
Nicotine use days, mean (SD)	3.19 (3.26)	2.60 (3.23)	*p=* 0.146
**Substance use disorder diagnoses, No. (%)**			
Suggestive nicotine dependence^c^	65 (50.39)	31 (49.21)	*p=* 1
Alcohol use disorder^a^	69 (53.49)	53 (84.13)	*p* < 0.001
Stimulant use disorder^a^	9 (6.98)	-	*p=* 0.074
Cocaine use disorder^a^	12 (9.30)	2 (3.17)	*p=* 0.216
Opioid use disorder^a^	2 (1.55)	-	*p=* 0.813
Hallucinogen use disorder^a^	11 (8.53)	1 (1.59)	*p=* 0.122
Inhalant use disorder^a^	3 (2.33)	-	*p=* 0.548
Sedative use disorder^a^	1 (0.78)	-	*p=* 1

a Determined using Mini International Neuropsychiatric Interview (MINI). Current diagnosis was used to reflect conditions at time of visit. Some participants have multiple SUDs.

b Calculated using the Timeline Followback for the seven days prior to saliva collection.

c Defined as a score of 6 or above on the Modified Version of the Fagerstrom Tolerance Questionnaire (mFTQ) and 4 or above on the Penn State Electronic Cigarette Dependence Index (PSECDI).

## Data Availability

The datasets analyzed during the current study are available in the NCBI Sequence Read Archive (SRA), under accession number PRJNA1238848.

## Appendix A. Supporting information

Supplementary data associated with this article can be found in the online version at doi:10.1016/j.drugalcdep.2025.112822.

## References

[R1] Adil NA, Omo-Erigbe C, Yadav H, Jain S, 2025. The oral–gut microbiome–brain axis in cognition. Microorganisms 13 (4), 814. 40284650 10.3390/microorganisms13040814 PMC12029813

[R2] Ames NJ, J BJ, Kornel S, Sarah M, K MB, S TRT, T BA, Narjis K, Shanna Y, Kelly R, Nancy D, Michael K, R WG, Goldman D, 2020. Longitudinal gut microbiome changes in alcohol use disorder are influenced by abstinence and drinking quantity. Gut Microbes 11 (6), 1608–1631. 10.1080/19490976.2020.1758010. 32615913 PMC7527072

[R3] Anderson MJ, 2017. Permutational multivariate analysis of variance (PERMANOVA). Wiley StatsRef Stat. Ref Online 1–15. 10.1002/9781118445112.stat07841.

[R4] Aqawi M, Sionov RV, Gallily R, Friedman M, Steinberg D, 2021. Anti-Bacterial properties of cannabigerol toward streptococcus mutans. Front. Microbiol 12, 656471. 10.3389/fmicb.2021.656471. 33967995 PMC8100047

[R5] Askari MS, Keyes KM, Mauro PM, 2021. Cannabis use disorder treatment use and perceived treatment need in the United States: time trends and age differences between 2002 and 2019. Drug Alcohol Depend. 229, 109154. 10.1016/j.drugalcdep.2021.109154. 34741874 PMC8671260

[R6] Austin PC, Stuart EA, 2015. Moving towards best practice when using inverse probability of treatment weighting (IPTW) using the propensity score to estimate causal treatment effects in observational studies. Stat. Med 34 (28), 3661–3679. 10.1002/sim.6607. 26238958 PMC4626409

[R7] Baker NL, Gray KM, Sherman BJ, Morella K, Sahlem GL, Wagner AM, McRae-Clark AL, 2018. Biological correlates of self-reported new and continued abstinence in cannabis cessation treatment clinical trials. Drug Alcohol Depend. 187, 270–277. 10.1016/j.drugalcdep.2018.03.017. 29698894 PMC5959795

[R8] Blaskovich MAT, Kavanagh AM, Elliott AG, Zhang B, Ramu S, Amado M, Lowe GJ, Hinton AO, Pham DMT, Zuegg J, Beare N, Quach D, Sharp MD, Pogliano J, Rogers AP, Lyras D, Tan L, West NP, Crawford DW, Thurn M, 2021. The antimicrobial potential of cannabidiol. Commun. Biol 4 (1), 7. 10.1038/s42003-020-01530-y. 33469147 PMC7815910

[R9] Bowland GB, Weyrich LS, 2022. The Oral-Microbiome-Brain axis and neuropsychiatric disorders: an anthropological perspective [Review]. Front. Psychiatr 13. 〈https://www.frontiersin.org/journals/psychiatry/articles/10.3389/fpsyt.2022.810008〉. 10.3389/fpsyt.2022.810008 PMC9005879 35432038

[R10] Browning BD, Kirkland AE, Green R, Engevik M, Alekseyenko AV, Leggio L, Tomko RL, Squeglia LM, 2024. The adolescent and young adult microbiome and its association with substance use: a scoping review. Alcohol Alcohol 59 (1). 10.1093/alcalc/agad055. PMC10979412 37665023

[R11] Cebrián G, Condón S, Mañas P, 2017. Physiology of the inactivation of vegetative bacteria by thermal treatments: mode of action, influence of environmental factors and inactivation kinetics. Foods 6 (12). 10.3390/foods6120107. PMC5742775 29189748

[R12] Chandra S, Radwan MM, Majumdar CG, Church JC, Freeman TP, ElSohly MA, 2019. New trends in cannabis potency in USA and Europe during the last decade (2008–2017). Eur. Arch. Psychiatr. Clin. Neurosci 269 (1), 5–15. 10.1007/s00406-019-00983-5. 30671616

[R13] Chen T, Yu W-H, Izard J, Baranova OV, Lakshmanan A, Dewhirst FE, 2010. The human oral microbiome database: a web accessible resource for investigating oral microbe taxonomic and genomic information. Database 2010, baq013. 10.1093/database/baq013. 20624719 PMC2911848

[R14] Cyrus E, Coudray MS, Kiplagat S, Mariano Y, Noel I, Galea JT, Hadley D, Dévieux JG, Wagner E, 2021. A review investigating the relationship between cannabis use and adolescent cognitive functioning. Curr. Opin. Psychol 38, 38–48. 10.1016/j.copsyc.2020.07.006. 32818908 PMC7365113

[R15] Davis CN, Markowitz JS, Squeglia LM, Ellingson JM, McRae-Clark AL, Gray KM, Kretschmer D, Tomko RL, 2024. Evidence for sex differences in the impact of cytochrome P450 genotypes on early subjective effects of cannabis. Addict. Behav 153, 107996. 10.1016/j.addbeh.2024.107996. 38394959 PMC10947802

[R16] Diagnostic and statistical manual of mental disorders: DSM-5^™^, 5th ed, American Psychiatric Publishing, Inc. xliv, 947–xliv, 947 (2013).

[R17] ElSohly MA, Chandra S, Radwan M, Majumdar CG, Church JC, 2021. A comprehensive review of cannabis potency in the United States in the last decade. Biol. Psychiatr. Cogn. Neurosci. Neuroimaging 6 (6), 603–606. 10.1016/j.bpsc.2020.12.016. 33508497

[R18] Fischer AS, Tapert SF, Louie DL, Schatzberg AF, Singh MK, 2020. Cannabis and the developing adolescent brain. Curr. Treat. Options Psychiatr 7 (2), 144–161. 10.1007/s40501-020-00202-2. PMC7380653 32714742

[R19] Foulds J, Veldheer S, Yingst J, Hrabovsky S, Wilson SJ, Nichols TT, Eissenberg T, 2015. Development of a questionnaire for assessing dependence on electronic cigarettes among a large sample of ex-smoking E-cigarette users. Nicotine Tob. Res 17 (2), 186–192. 10.1093/ntr/ntu204. 25332459 PMC4838001

[R20] Freeman TP, Craft S, Wilson J, Stylianou S, ElSohly M, Di Forti M, Lynskey MT, 2021. Changes in delta-9-tetrahydrocannabinol (THC) and cannabidiol (CBD) concentrations in cannabis over time: systematic review and meta-analysis. Addiction 116 (5), 1000–1010. 10.1111/add.15253. 33160291

[R21] Greifer N, 2025. Weight. Weight. Covariate Balance Obs. Stud 〈https://ngreifer.github.io/WeightIt/〉.

[R22] Gryka-Marton M, Grabowska AD, Szukiewicz D, 2025. Breaking the barrier: the role of proinflammatory cytokines in BBB dysfunction. Int J. Mol. Sci 26 (8). 10.3390/ijms26083532. PMC12026921 40331982

[R23] Gualtero DF, Lafaurie GI, Buitrago DM, Castillo Y, Vargas-Sanchez PK, Castillo DM, 2023. Oral microbiome mediated inflammation, a potential inductor of vascular diseases: a comprehensive review [Review]. Front. Cardiovasc. Med 10, 2023. 〈https://www.frontiersin.org/journals/cardiovascular-medicine/articles/10.3389/fcvm.2023.1250263〉. 10.3389/fcvm.2023.1250263 PMC10498784 37711554

[R24] Gunn CM, Pankowska M, Harris M, Helsing E, Battaglia TA, Bagley SM, 2022. The representation of females in clinical trials for substance use disorder conducted in the United States (2010–19). Addiction 117 (10), 2583–2590. 10.1111/add.15842. 35165969 PMC10062729

[R25] Ha JY, Seok J, Kim S-J, Jung H-J, Ryu K-Y, Nakamura M, Jang I-S, Hong S-H, Lee Y, Lee H-J, 2023. Periodontitis promotes bacterial extracellular vesicle-induced neuroinflammation in the brain and trigeminal ganglion. PLOS Pathog. 19 (10), e1011743. 10.1371/journal.ppat.1011743. 37871107 PMC10621956

[R26] Hayashi Y, Saito T, Ohshima T, Nakagawa Y, Maeda N, 2015. Alterations of the oral microbiota and oral clinical findings in dry mouth. J. Oral. Biosci 57 (4), 171–174. 10.1016/j.job.2015.07.001.

[R27] John WS, Zhu H, Mannelli P, Schwartz RP, Subramaniam GA, Wu L-T, 2018. Prevalence, patterns, and correlates of multiple substance use disorders among adult primary care patients. Drug Alcohol Depend. 187, 79–87. 10.1016/j.drugalcdep.2018.01.035. 29635217 PMC5959766

[R28] Kageyama S, Sakata S, Ma J, Asakawa M, Takeshita T, Furuta M, Ninomiya T, Yamashita Y, 2023. High-Resolution detection of translocation of oral bacteria to the gut. J. Dent. Res 102 (7), 752–758. 10.1177/00220345231160747. 37204134 PMC10288163

[R29] Kohno M, Link J, Dennis LE, McCready H, Huckans M, Hoffman WF, Loftis JM, 2019. Neuroinflammation in addiction: a review of neuroimaging studies and potential immunotherapies. Pharm. Biochem. Behav 179, 34–42. 10.1016/j.pbb.2019.01.007. PMC6637953 30695700

[R30] Kosciolek T, Victor TA, Kuplicki R, Rossi M, Estaki M, Ackermann G, Knight R, Paulus MP, 2021. Individuals with substance use disorders have a distinct oral microbiome pattern. Brain Behav. Immun. Health 15, 100271. 10.1016/j.bbih.2021.100271. 34589776 PMC8474247

[R31] Lawn W, Mokrysz C, Lees R, Trinci K, Petrilli K, Skumlien M, Borissova A, Ofori S, Bird C, Jones G, Bloomfield MAP, Das RK, Wall MB, Freeman TP, Curran HV, 2022. The CannTeen study: cannabis use disorder, depression, anxiety, and psychotic-like symptoms in adolescent and adult cannabis users and age-matched controls. J. Psychopharmacol 36 (12), 1350–1361. 10.1177/02698811221108956. 35772419 PMC9716489

[R32] Leinen ZJ, Mohan R, Premadasa LS, Acharya A, Mohan M, Byrareddy SN, 2023. Therapeutic potential of cannabis: a comprehensive review of current and future applications. Biomedicines 11 (10). 10.3390/biomedicines11102630. PMC10604755 37893004

[R33] Liu C, Qi X, Yang D, Neely A, Zhou Z, 2020. The effects of cannabis use on oral health. Oral. Dis 26 (7), 1366–1374. 10.1111/odi.13246. 31793130

[R34] Lundtorp-Olsen C, Enevold C, Juel Jensen CA, Stofberg SN, Twetman S, Belstrøm D, 2021. Impact of probiotics on the salivary microbiota and salivary levels of Inflammation-Related proteins during Short-Term sugar stress: a randomized controlled trial. Pathogens 10 (4). 10.3390/pathogens10040392 PMC8064398 33805894

[R35] Luo Z, Fitting S, Robinson C, Benitez A, Li M, Wu Y, Fu X, Amato D, Ning W, Funderburg N, Wang X, Zhou Z, Yu X, Wagner A, Cong X, Xu W, Maas K, Wolf BJ, Huang L, Jiang W, 2021. Chronic cannabis smoking-enriched oral pathobiont drives behavioral changes, macrophage infiltration, and increases β-amyloid protein production in the brain. eBioMedicine 74, 103701. 10.1016/j.ebiom.2021.103701. 34826801 PMC8626580

[R36] Maitre Y, Micheneau P, Delpierre A, Mahalli R, Guerin M, Amador G, Denis F, 2020. Did the brain and oral microbiota talk to each other? A review of the literature. J. Clin. Med 9 (12). 10.3390/jcm9123876 PMC7760025 33260581

[R37] Mallick H, Rahnavard A, McIver LJ, Ma S, Zhang Y, Nguyen LH, Tickle TL, Weingart G, Ren B, Schwager EH, Chatterjee S, Thompson KN, Wilkinson JE, Subramanian A, Lu Y, Waldron L, Paulson JN, Franzosa EA, Bravo HC, Huttenhower C, 2021. Multivariable association discovery in population-scale meta-omics studies. PLoS Comput. Biol 17 (11), e1009442. 10.1371/journal.pcbi.1009442. 34784344 PMC8714082

[R38] McGrath JJ, Al-Hamzawi A, Alonso J, Altwaijri Y, Andrade LH, Bromet EJ, Bruffaerts R, de Almeida JMC, Chardoul S, Chiu WT, Degenhardt L, Demler OV, Ferry F, Gureje O, Haro JM, Karam EG, Karam G, Khaled SM, Kovess-Masfety V, Zaslavsky AM, 2023. Age of onset and cumulative risk of mental disorders: a cross-national analysis of population surveys from 29 countries. Lancet Psychiatr. 10 (9), 668–681. 10.1016/S2215-0366(23)00193-1. PMC10529120 37531964

[R39] McMurdie PJ, Holmes S, 2013. Phyloseq: an r package for reproducible interactive analysis and graphics of microbiome census data. PLoS One 8 (4), e61217. 10.1371/journal.pone.0061217. 23630581 PMC3632530

[R40] Mohammed LI, Razali R, Zakaria ZZ, Benslimane FM, Cyprian F, Al-Asmakh M, 2024. Smoking induced salivary microbiome dysbiosis and is correlated with lipid biomarkers. BMC Oral. Health 24 (1), 608. 10.1186/s12903-024-04340-4. 38796419 PMC11127352

[R41] Narengaowa, Kong W, Lan F, Awan UF, Qing H, Ni J, 2021. The Oral-Gut-Brain AXIS: the influence of microbes in Alzheimer’s disease [Review]. Front. Cell. Neurosci 15. 10.3389/fncel.2021.633735. PMC8079629 33935651

[R42] Newman T, Krishnan LP, Lee J, Adami GR, 2019. Microbiomic differences at cancer-prone oral mucosa sites with marijuana usage. Sci. Rep 9 (1), 12697. 10.1038/s41598-019-48768-z. 31481657 PMC6722050

[R43] Ogunrinola GA, Oyewale JO, Oshamika OO, Olasehinde GI, 2020. The human microbiome and its impacts on health. Int. J. Microbiol 2020 (1), 8045646. 10.1155/2020/8045646. 32612660 PMC7306068

[R44] Oksanen J, Simpson GL, Blanchet FG, Kindt R, Legendre P, Minchin PR, O’Hara RB, Solymos P, Stevens MHH, Szoecs E, Wagner H, Barbour M, Bedward M, Bolker B, Borcard D, Carvalho G, Chirico M, Caceres MD, Durand S, Weedon J, 2024. Vegan Community Ecol. Package. 〈https://vegandevs.github.io/vegan/〉.

[R45] Panee J, Gerschenson M, Chang L, 2018. Associations between microbiota, mitochondrial function, and cognition in chronic marijuana users. J. Neuroimmune Pharmacol 13 (1), 113–122. 10.1007/s11481-017-9767-0. 29101632 PMC5790619

[R46] Panee J, Qin Y, Deng Y, 2024. Associations of chronic marijuana use with changes in salivary microbiome. Microorganisms 12 (11). 10.3390/microorganisms12112244. PMC11596347 39597633

[R47] Prokhorov AV, De Moor C, Pallonen UE, Suchanek Hudmon K, Koehly L, Hu S, 2000. Validation of the modified fagerström tolerance questionnaire with salivary cotinine among adolescents. Addict. Behav 25 (3), 429–433. 10.1016/S0306-4603(98)00132-4. 10890296

[R48] Prokhorov AV, Pallonen UE, Fava JL, Ding L, Niaura R, 1996. Measuring nicotine dependence among high-risk adolescent smokers. Addict. Behav 21 (1), 117–127. 10.1016/0306-4603(96)00048-2. 8729713

[R49] R Core Team, 2023. R: a language and environment for statistical computing. R. Found. Stat. Comput. 〈https://www.R-project.org/〉.

[R50] Riviere GR, Riviere KH, Smith KS, 2002. Molecular and immunological evidence of oral treponema in the human brain and their association with alzheimer’s disease. Oral. Microbiol. Immunol 17 (2), 113–118. 10.1046/j.0902-0055.2001.00100.x. 11929559

[R51] Rosenbaum PR, Rubin DB, 1983. The central role of the propensity score in observational studies for causal effects. Biometrika 70 (1), 41–55. 10.1093/biomet/70.1.41.

[R52] Schmidt TSB, Hayward MR, Coelho LP, Li SS, Costea PI, Voigt AY, Wirbel J, Maistrenko OM, Alves RJC, Bergsten E, de Beaufort C, Sobhani I, Heintz-Buschart A, Sunagawa S, Zeller G, Wilmes P, Bork P, 2019. Extensive transmission of microbes along the gastrointestinal tract. eLife 8, e42693. 10.7554/eLife.42693. 30747106 PMC6424576

[R53] Sheehan DV, Lecrubier Y, Sheehan KH, Amorim P, Janavs J, Weiller E, Hergueta T, Baker R, Dunbar GC, 1998. The Mini-International neuropsychiatric interview (M.I.N.I.): the development and validation of a structured diagnostic psychiatric interview for DSM-IV and ICD-10. J. Clin. Psychiatr 59 (20), 34–57, 22–33;quiz. 9881538

[R54] Sherman BJ, McRae-Clark AL, 2016. Treatment of cannabis use disorder: current science and future outlook. Pharmacotherapy 36 (5), 511–535. 10.1002/phar.1747. 27027272 PMC4880536

[R55] Shetty L.L. a S., 2012-2019. Micro R. Package.

[R56] Siddiqui H, Eribe Ribs EK, Singhrao SK, Olsen I, 2019. High throughput sequencing detects gingivitis and periodontal oral bacteria in alzheimer’s disease autopsy brains. J. Neurosci. Res 1 (1), 3.

[R57] Sobell LC, Sobell MB, 1992. Timeline follow-back: A technique for assessing self-reported alcohol consumption. Measuring alcohol consumption: Psychosocial and biochemical methods. Humana Press/Springer Nature, pp. 41–72. 10.1007/978-1-4612-0357-5_3.

[R58] Substance Abuse and Mental Health Services Administration (SAMHSA), 2024. Key Subst. Use Ment. Health Indic. U. S. Results 2023 Natl. Surv. Drug Use Health. 〈https://www.samhsa.gov/data/〉.

[R59] Tao K, Yuan Y, Xie Q, Dong Z, 2024. Relationship between human oral microbiome dysbiosis and neuropsychiatric diseases: an updated overview. Behav. Brain Res 471, 115111. 10.1016/j.bbr.2024.115111. 38871130

[R60] Thomson WM, Poulton R, Broadbent JM, Moffitt TE, Caspi A, Beck JD, Welch D, Hancox RJ, 2008. Cannabis smoking and periodontal disease among young adults. Jama 299 (5), 525–531. 10.1001/jama.299.5.525. 18252882 PMC2823391

[R61] Wickham H, 2016. ggplot2: Elegant graphics for data analysis. Springer-Verlag, New York. 〈https://ggplot2.tidyverse.org〉.

[R62] Wu J, Peters BA, Dominianni C, Zhang Y, Pei Z, Yang L, Ma Y, Purdue MP, Jacobs EJ, Gapstur SM, Li H, Alekseyenko AV, Hayes RB, Ahn J, 2016. Cigarette smoking and the oral microbiome in a large study of American adults. ISME J. 10 (10), 2435–2446. 10.1038/ismej.2016.37. 27015003 PMC5030690

[R63] Zaura E, Brandt Bernd W, Teixeira de Mattos MJ, Buijs Mark J, Caspers Martien PM, Rashid M-U, Weintraub A, Nord Carl E, Savell A, Hu Y, Coates Antony R, Hubank M, Spratt David A, Wilson M, Keijser Bart JF, Crielaard W, 2015. Same exposure but two radically different responses to antibiotics: resilience of the salivary microbiome versus Long-Term microbial shifts in feces, 10.1128/mbio.01693-01615 mBio 6 (6). https://doi.org/10.1128/mbio.01693-15. PMC4659469 26556275

[R64] Zaura E, Pappalardo VY, Buijs MJ, Volgenant CMC, Brandt BW, 2021. Optimizing the quality of clinical studies on oral microbiome: a practical guide for planning, performing, and reporting. Periodontol 2000 85 (1), 210–236. 10.1111/prd.12359. 33226702 PMC7756869

