# Supplementary material for: Oral microbial profiles in young adults with cannabis use disorder
Source: Drug Alcohol Depend. Author manuscript; Available in PMC 2026 Apr 21. (PMC13098379; doi:10.1016/j.drugalcdep.2025.112822)
Supplement: Appendix A. Supplementary material [file NIHMS2162595-supplement-Appendix_A__Supplementary_material.docx]

Supplementary Materials


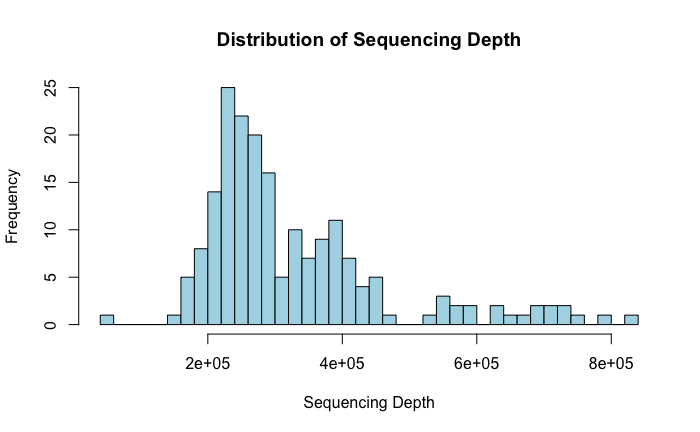


**Figure S1. Distribution of Sequencing Depth.**


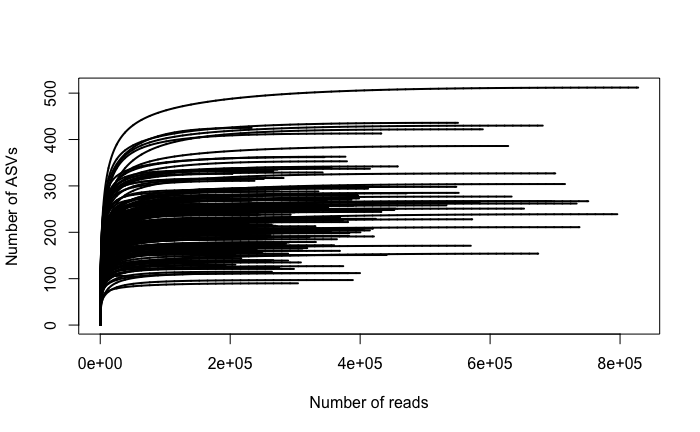


**Figure S2. Rarefaction curve.**

**Saliva gDNA Isolation Protocol:**

1. Transfer 500 ul of saliva into Eppendorf tubes
2. Add 250 ul of lysis (ASL) buffer to the saliva
3. Incubate at 60 degrees Celsius for 30 minutes
4. Move entire sample into tubes with .1 beads
5. Homogenize for 2x 20 seconds cycles on fastprep bead beater system
6. After bead beating, centrifuge the 2mL tubes for 5 minutes at 16,000x g
7. Transfer the supernatant to the labeled Eppendorf tubes to pool the sample
8. Total volume = 500 ul
9. Add 500 ul of 100% ETOH
10. Add 500 ul lysate to Epoch Biosciences gDNA spin columns
11. Close the cap and centrifuge at 12,000 g for 1 minute
12. Discard the flow through
13. Add remaining 500 ul lysate to Epoch Biosciences gDNA spin columns
14. Close the cap and centrifuge at 12,000 g for 1 minute
15. Discard the flow through
16. Add 500 ul AW1
17. Close the cap and centrifuge at 12,000 g for 1 minute
18. Discard the flow through
19. Add 500 ul Buffer AW2
20. Close the cap and centrifuge at 12,000 rpm for 1 minute
21. Discard the flow through
22. Transfer the spin columns into new labeled 1.5 mL microcentrifuge tube
23. Carefully open the spin column and pipet 200 ul Buffer AE directly onto the spin column membrane
24. Close the cap and incubate for 1 minute at room temperature
25. Centrifuge at 12,000 g for 1 minute to elute DNA
26. Store at -20 degrees Celsius


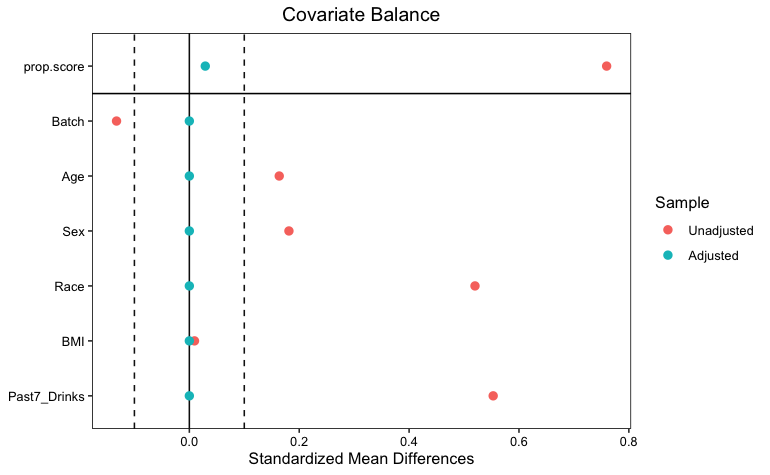


**Figure S3. Love plot showing balancing covariates before and after propensity score matching at the genus level.**


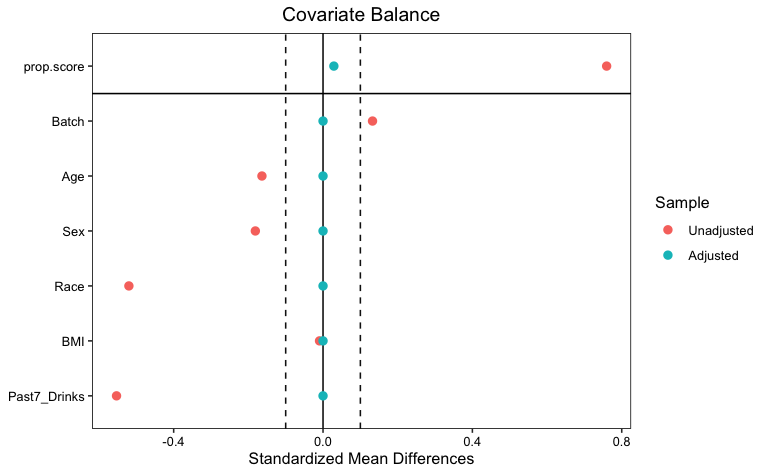


**Figure S4. Love plot showing balancing covariates before and after propensity score matching at the species level.**


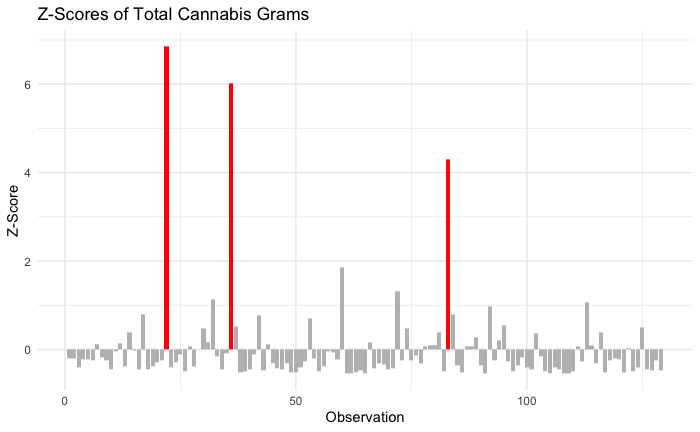


**Figure S5. Z-scores of total cannabis grams.**


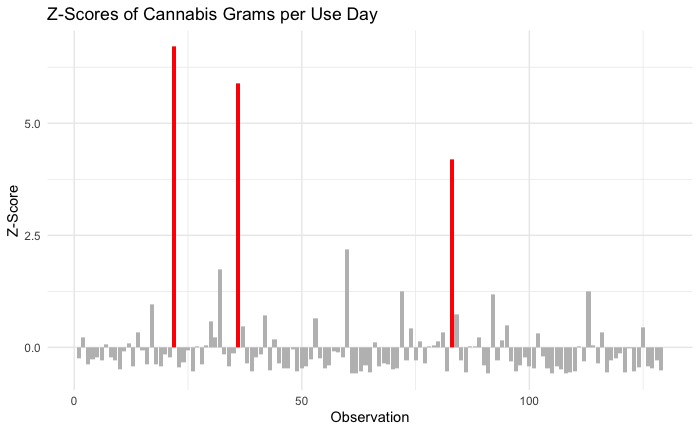


**Figure S6. Z-Scores of cannabis grams per use day.**

**Table S1. Aim 1 Alpha Diversity Genus**

| **Taxa level** | **Index** | **Estimate** | **Std. Error** | **Statistic** | **P value** | **2.50%** | **97.50%** |
| --- | --- | --- | --- | --- | --- | --- | --- |
| Genus | Shannon | -0.198 | 0.043 | -4.621 | **0.000** | -0.282 | -0.114 |
|  | Simpson | -0.014 | 0.005 | -2.913 | **0.004** | -0.023 | -0.005 |
|  | Chao1 | -2.914 | 1.778 | -1.639 | 0.101 | -6.400 | 0.571 |
|  | Pielou’s evenness | -0.042 | 0.009 | -4.639 | **0.000** | -0.060 | -0.024 |
| Species | Shannon | -0.254 | 0.064 | -3.974 | **0.000** | -0.379 | -0.129 |
|  | Simpson | -0.009 | 0.003 | -2.579 | **0.010** | -0.016 | -0.002 |
|  | Chao1 | -9.996 | 6.212 | -1.609 | 0.108 | -22.171 | 2.179 |
|  | Pielou’s evenness | -0.043 | 0.010 | -4.250 | **0.000** | -0.063 | -0.023 |

**Table S2. Aim 1 Beta Diversity**

| **Taxa level** | **Metric** | **Sum of Sqs** | **R2** | **F** | **P value** |
| --- | --- | --- | --- | --- | --- |
| Genus | Bray-Curtis | 0.254 | 0.001 | 1.967 | 0.054 |
|  | Jaccard | 0.383 | 0.006 | 1.252 | 0.167 |
|  | Aitchison | 862 | 0.013 | 2.620 | **0.001** |
| Species | Bray-Curtis | 0.303 | 0.001 | 1.746 | 0.058 |
|  | Jaccard | 0.421 | 0.001 | 1.203 | 0.109 |
|  | Aitchison | 3074 | 0.011 | 2.152 | **0.001** |

**Table S3. Aim 2 Alpha Diversity**

| **Variable** | **Taxa level** | **Index** | **Estimate** | **Std. Error** | **Statistic** | **P value** | **2.50%** | **97.50%** |
| --- | --- | --- | --- | --- | --- | --- | --- | --- |
| Days | Genus | Shannon | -0.015 | 0.017 | -0.851 | 0.397 | -0.049 | 0.020 |
|  |  | Simpson | 0.001 | 0.002 | 0.626 | 0.532 | -0.002 | 0.004 |
|  |  | Chao1 | -1.432 | 0.572 | -2.506 | **0.014** | -2.564 | -0.300 |
|  |  | Pielou’s evenness | -0.001 | 0.004 | -0.206 | 0.837 | -0.008 | 0.007 |
|  | Species | Shannon | -0.024 | 0.025 | -0.980 | 0.329 | -0.074 | 0.025 |
|  |  | Simpson | 0.001 | 0.001 | 0.744 | 0.458 | -0.001 | 0.003 |
|  |  | Chao1 | -4.848 | 1.948 | -2.489 | **0.014** | -8.706 | -0.991 |
|  |  | Pielou’s evenness | -0.002 | 0.004 | -0.376 | 0.708 | -0.010 | 0.007 |
| Grams per day | Genus | Shannon | -0.014 | 0.019 | -0.766 | 0.445 | -0.051 | 0.022 |
|  |  | Simpson | -0.002 | 0.002 | -1.503 | 0.135 | -0.006 | 0.001 |
|  |  | Chao1 | 0.538 | 0.623 | 0.863 | 0.390 | -0.696 | 1.771 |
|  |  | Pielou’s evenness | -0.004 | 0.004 | -1.039 | 0.301 | -0.012 | 0.004 |
|  | Species | Shannon | -0.005 | 0.027 | -0.178 | 0.859 | -0.057 | 0.048 |
|  |  | Simpson | -0.001 | 0.001 | -0.656 | 0.513 | -0.003 | 0.002 |
|  |  | Chao1 | 1.025 | 2.126 | 0.482 | 0.631 | -3.185 | 5.236 |
|  |  | Pielou’s evenness | -0.001 | 0.005 | -0.286 | 0.775 | -0.011 | 0.008 |
| Total grams | Genus | Shannon | -0.002 | 0.003 | -0.854 | 0.395 | -0.008 | 0.003 |
|  |  | Simpson | 0.000 | 0.000 | -1.538 | 0.127 | -0.001 | 0.000 |
|  |  | Chao1 | 0.070 | 0.098 | 0.714 | 0.477 | -0.124 | 0.265 |
|  |  | Pielou’s evenness | -0.001 | 0.001 | -1.106 | 0.271 | -0.002 | 0.001 |
|  | Species | Shannon | -0.001 | 0.004 | -0.256 | 0.798 | -0.009 | 0.007 |
|  |  | Simpson | 0.000 | 0.000 | -0.498 | 0.619 | 0.000 | 0.000 |
|  |  | Chao1 | 0.084 | 0.335 | 0.250 | 0.803 | -0.580 | 0.747 |
|  |  | Pielou’s evenness | 0.000 | 0.001 | -0.323 | 0.747 | -0.002 | 0.001 |

**Table S4. Aim 2 Beta Diversity**

| **Variable** | **Taxa level** | **Metric** | **Sum of Sqs** | **R2** | **F** | **P value** |
| --- | --- | --- | --- | --- | --- | --- |
| Days | Genus | Bray-Curtis | 0.131 | 0.008 | 1.005 | 0.420 |
|  |  | Jaccard | 0.302 | 0.008 | 0.982 | 0.468 |
|  |  | Aitchison | 380.000 | 0.009 | 1.198 | 0.200 |
|  | Species | Bray-Curtis | 0.158 | 0.007 | 0.922 | 0.497 |
|  |  | Jaccard | 0.346 | 0.008 | 0.994 | 0.461 |
|  |  | Aitchison | 3315.000 | 0.012 | 2.323 | **0.001** |
| Grams per day | Genus | Bray-Curtis | 0.063 | 0.004 | 0.458 | 0.858 |
|  |  | Jaccard | 0.273 | 0.007 | 0.885 | 0.613 |
|  |  | Aitchison | 324.000 | 0.008 | 1.022 | 0.277 |
|  | Species | Bray-Curtis | 0.155 | 0.007 | 0.902 | 0.516 |
|  |  | Jaccard | 0.363 | 0.008 | 1.043 | 0.342 |
|  |  | Aitchison | 1711.000 | 0.006 | 1.192 | 0.166 |
| Total grams | Genus | Bray-Curtis | 0.071 | 0.004 | 0.539 | 0.807 |
|  |  | Jaccard | 0.286 | 0.007 | 0.928 | 0.532 |
|  |  | Aitchison | 302.000 | 0.007 | 0.950 | 0.471 |
|  | Species | Bray-Curtis | 0.180 | 0.008 | 1.051 | 0.378 |
|  |  | Jaccard | 0.389 | 0.009 | 1.116 | 0.219 |
|  |  | Aitchison | 1677.000 | 0.006 | 1.168 | 0.179 |
